# Bilirrubina: Medición y utilidad clínica en la enfermedad hepática

**DOI:** 10.1515/almed-2021-0016

**Published:** 2021-05-20

**Authors:** Armando Raúl Guerra-Ruiz, Javier Crespo, Rosa Maria López Martínez, Paula Iruzubieta, Gregori Casals Mercadal, Marta Lalana Garcés, Bernardo A. Lavin Gomez, Manuel Morales Ruiz

**Affiliations:** Servicio de Análisis Clínicos, Hospital Universitario Marqués de Valdecilla, Santander, España; Comisión de Valoración Bioquímica de la Enfermedad Hepática, SEQC^ML^ , Barcelona, España; Servicio de Bioquímica y Genética Molecular, Hospital Clínic de Barcelona, IDIBAPS, CIBEReh, Barcelona, España; Servicio de Análisis Clínicos, Hospital de Barbastro, Huesca, España; Unidad de Patología hepática, Departamentos de Bioquímica y Microbiología, Hospital Universitari Vall d’Hebron, Universitat Autònoma de Barcelona, Barcelona, España; Departamento de Biomedicina de la Facultad de Medicina y Ciencias de la Salud, Universidad de Barcelona, Barcelona, España; Servicio Aparato Digestivo, Hospital Universitario Marqués de Valdecilla, Santander, España; Grupo de Investigación Clínica y Traslacional en Enfermedades Digestivas, IDIVAL, Santander, España; Sociedad Española de Patología Digestiva (SEPD), Madrid, España

**Keywords:** bilirrubina, enfermedades hepáticas, biomarcador, hepatopatía, colestasis, método diazo

## Abstract

Un aumento en los niveles plasmáticos de bilirrubina es una alteración frecuente. Puede deberse a cualquier causa que altere alguna de las fases de su metabolismo: a) producción excesiva de bilirrubina (ej. hemólisis patológica); b) defecto en la captación hepática, con aumento de bilirrubina indirecta); c) defecto de conjugación, por alteración del enzima encargada (UDP-glucuronosiltransferasa); y d) defecto de excreción biliar, con aumento de bilirrubina directa, por defectos en las proteínas encargadas de la excreción, o bien por la imposibilidad del paso de la bilis a través de los conductos biliares hasta el intestino. Una lesión hepática de cualquier causa, al disminuir el número de hepatocitos, puede producir una disminución de la captación de bilirrubina indirecta desde el plasma y una disminución del transporte y excreción de la bilirrubina directa hacia los conductillos biliares. Se pueden usar diferentes técnicas analíticas para medir la bilirrubina y sus metabolitos en el suero, la orina y las heces. La bilirrubina sérica se mide mediante (1) la "reacción diazo", actualmente el método de referencia; (2) cromatografía líquida de alta resolución (HPLC); (3) métodos oxidativos, enzimáticos y químicos; (4) espectrofotometría directa; y (5) métodos transcutáneos. Aunque la bilirrubina es un marcador clásico de disfunción hepática, no siempre indica una lesión de este órgano. Por tanto, para obtener un diagnóstico preciso, el significado de las alteraciones de este parámetro biológico ha de valorarse en conjunción con la anamnesis del paciente, la magnitud de la alteración, y el patrón de las alteraciones bioquímicas. acompañantes.

## Introducción

La bilirrubina es un pigmento biliar de color amarillo anaranjado que resulta de la degradación del grupo hemo de varias proteínas, especialmente del catabolismo de la hemoglobina. El grupo hemo es degradado enzimáticamente liberando biliverdina, que es a su vez reducida a bilirrubina no conjugada (BNC) o indirecta, insoluble en agua, circulando en sangre ligada a albúmina. En el hígado, por medio de la adición de grupos glucurónido (conjugación) se transforma en hidrosoluble (bilirrubina directa) y es excretada a través de la bilis o, regresando a la circulación sanguínea, es filtrada por el riñón y excretada vía renal [1].

Un aumento en los niveles plasmáticos de bilirrubina es una alteración frecuente, observada tanto en la atención primaria [2] como en el ámbito hospitalario. Una lesión hepática de cualquier causa, al disminuir el número de hepatocitos, puede producir una hiperbilirrubinemia [3]. Puede deberse a cualquier causa que altere alguna de las fases de su metabolismo: producción excesiva, defecto en la captación hepática, defecto de su conjugación, o defecto de la excreción biliar [4].

La bilirrubina es un marcador clásico que es incluido rutinariamente dentro de los perfiles clínicos, tanto de pacientes con disfunción hepática como de pacientes con otras patologías. Sin embargo, no es un indicador sensible ni específico de este órgano y, por tanto, es necesario realizar una correcta interpretación de los resultados obtenidos con el objetivo de obtener un diagnóstico preciso. El significado de las alteraciones de este parámetro biológico ha de valorarse en conjunción con la anamnesis del paciente, la magnitud de la alteración, y las alteraciones bioquímicas acompañantes [5], [6]. En este contexto, el motivo de esta revisión es proporcionar herramientas para realizar una interpretación adecuada de las alteraciones séricas de la bilirrubina, y contextualizar su potencialidad en el diagnóstico diferencial de las enfermedades hepáticas.

## Bioquímica y metabolismo

**Figura 1:**
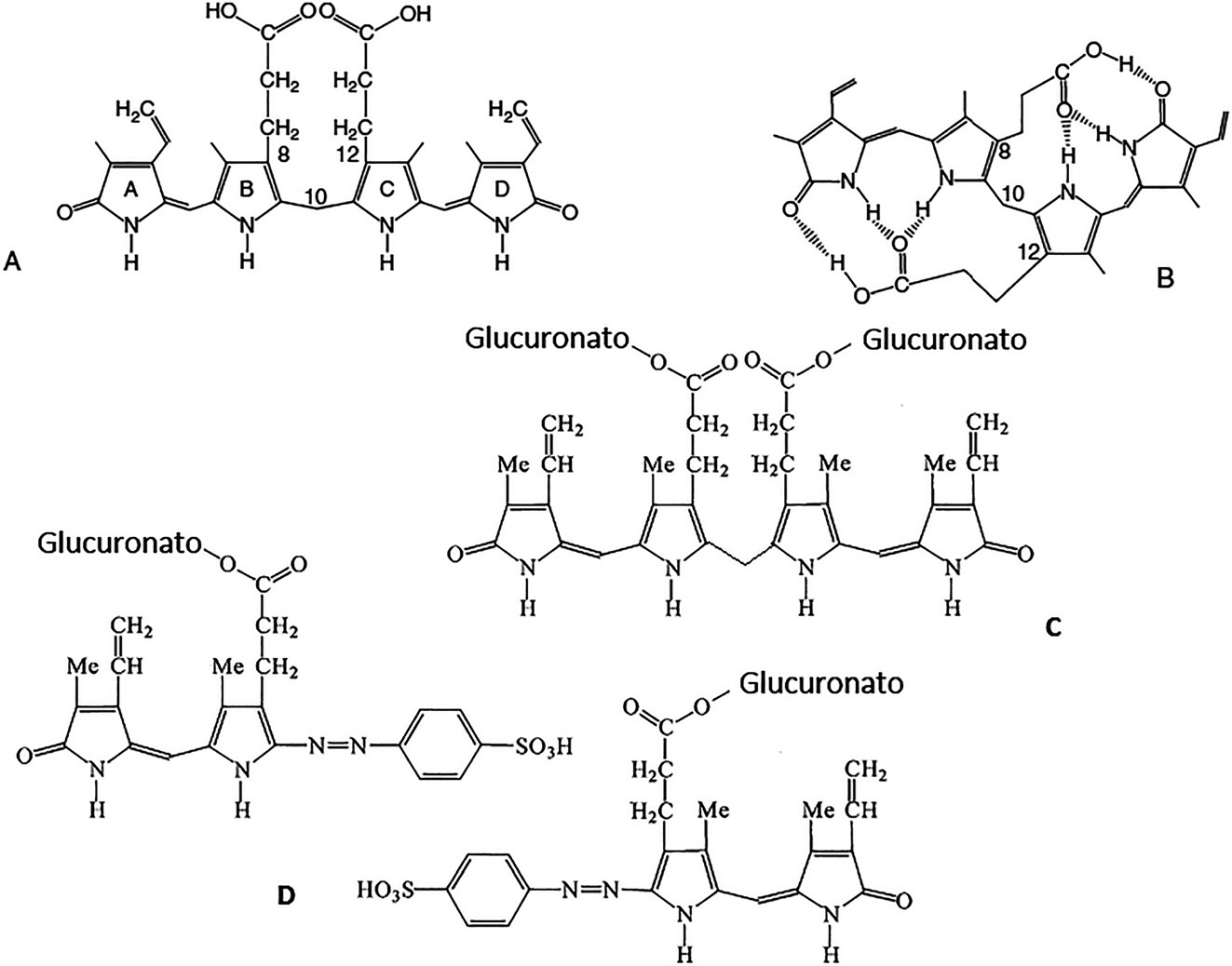
Bilirrubina, isómeros, y derivados. (A) Configuración lineal o abierta. (B) Configuración cerrada, cresta-apilada o mosaico. (C) Diglucurónido de bilirrubina (bilirrubina conjugada). (D) Azopirroles coloreados (azopigmentos) producto de la reacción diazo.

La bilirrubina consiste en una cadena abierta (lineal) de 4 anillos pirrólicos (Figura 1A), provenientes de la apertura del anillo tetrapirrólico de la protoporfirina (hemo). Esta estructura puede presentar varias formas isoméricas de las cuales la Bilirrubina IXa es la más abundante en vivo (cerca del 99%) [1]. Otros isómeros como IIIa y XIIIa son minoritarios en la circulación; el material de referencia de la bilirrubina SRM 916 del National Institute of Standards and Technology (NIST), el cual ya no está disponible, y otras preparaciones comerciales contienen cantidades sustanciales de ambos isómeros [7].

La bilirrubina se forma a partir del metabolismo del grupo hemo, mayormente de la hemoglobina procedente de la degradación de los hematíes envejecidos (80–85%); la fracción restante proviene de la hematopoyesis ineficaz, así como de otras proteínas que contienen un grupo hemo (mioglobina, citocromos y peroxidasas). El grupo hemo liberado, formado por una molécula de protoporfirina IX y un ion Fe^2+^, es degradado por la enzima hemo-oxigenasa [8]. para dar origen a una molécula lineal de 4 anillos pirrólicos llamada biliverdina; se genera además hierro libre (Fe^3+^) y monóxido de carbono. La biliverdina posteriormente es reducida por la enzima biliverdina reductasa para originar bilirrubina. Se forma mayoritariamente el isómero IXa, de configuración cerrada e hidrofóbica (Figura 1B) [9]. La unión de la bilirrubina a la albúmina (Kd≈10^−7^–10^−8^ mol/L) previene su isomerización y posibilita el traslado por la circulación hasta el hígado [1].

**Figura 2:**
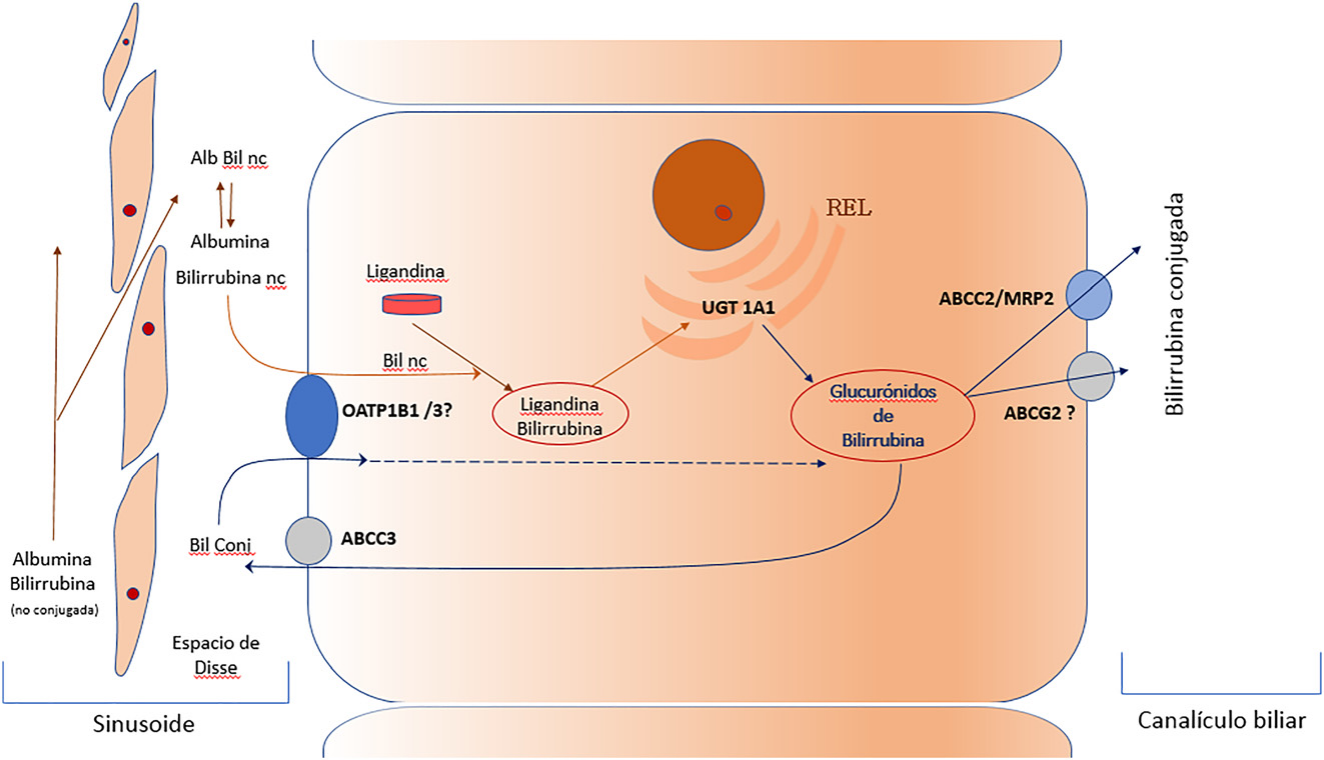
Captación, metabolismo y transporte de la bilirrubina por el hepatocito.Bil nc, bilirrubina no conjugada; Bil Conj, bilirrubina conjugada; REL, reticulo endoplásmico liso; UGT 1A1, uridina difosfato (UDP) -glucuroniltransferasa 1A1.

La bilirrubina unida a la albúmina penetra en el hígado por el polo sinusoidal. Las proteínas de transporte de aniones orgánicos (OATP) 1B1 y 1B3, codificadas en la superfamilia de genes transportadores de solutos-aniones orgánicos (SLCO), son responsables de la absorción de bilirrubina en el hepatocito [10], (Figura 2). Una vez dentro de las células hepáticas, la bilirrubina se une reversiblemente a proteínas solubles conocidas como ligandinas o proteínas Y, proteínas citosólicas de la familia de genes de la glutatión-S-transferasa, lo cual retarda el reflujo de esta de regreso al plasma [11]. Posteriormente, en el retículo endoplásmico liso, la bilirrubina se conjuga con el ácido glucurónico, por la acción de la UDPGT-1A1, para producir glucurónidos de bilirrubina [12]. El glucurónido de bilirrubina vuelve al citosol, donde se difundirá al polo canalicular para la secreción a la bilis, o al polo sinusoidal para su secreción al plasma, de donde es recaptada por los mismos transportadores OATP1B1/3 [6]. En el polo canalicular, el proceso está mediado por un transportador apical dependiente de ATP, ATP-binding-cassette-C2 (ABCC2), anteriormente denominado proteína relacionada con múltiples fármacos (MRP2–multidrug related-protein-2) [13].

**Figura 3:**
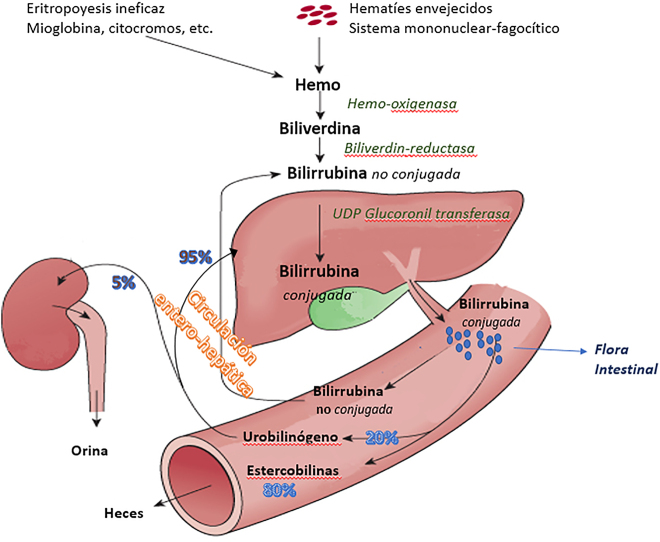
Metabolismo y recirculación de la bilirrubina.

La excreción de la bilirrubina conjugada (BC), ahora polar y soluble en agua, es un proceso de concentración dependiente de energía. Como resultado, la concentración de bilirrubina biliar es aproximadamente 100 veces mayor que la del citoplasma de los hepatocitos. La solubilidad en agua de la BC también contribuye a dificultar la reabsorción desde el tracto. Sin embargo, los monoglucurónidos y diglucurónidos de bilirrubina son compuestos relativamente inestables que se hidrolizan fácilmente a BNC. La acción de la β-glucuronidasa de las bacterias y de la mucosa intestinal contribuye a este efecto [14]. La bilirrubina revertida nuevamente a su estado no-conjugado puede absorberse fácilmente a través de la mucosa intestinal, regresando a la circulación enterohepática (Figura 3). Alrededor de un 25% de la bilirrubina excretada vía biliar sufre esta recirculación.

La mayor parte del resto de la BNC presente en el intestino es reducida por la flora microbiana intestinal anaeróbica para formar un grupo de tres tetrapirroles incoloros (estercobilinógeno, mesobilinógeno o urobilinógeno) denominados colectivamente urobilinógenos. En el tracto intestinal inferior, los tres urobilinógenos se oxidan espontáneamente para producir los pigmentos biliares correspondientes estercobilina, mesobilina y urobilina, de color marrón anaranjado, que constituyen los principales pigmentos de las heces. Hasta el 20% del urobilinógeno producido diariamente se reabsorbe del intestino y entra en la circulación enterohepática. La mayor parte del mismo es captada por el hígado (vía porta) y se re-excreta en la bilis; una pequeña fracción (2–5%) ingresa a la circulación general, se filtra por el riñón, y es detectable en la orina.

## Métodos analíticos

Se pueden usar diferentes técnicas analíticas para medir la bilirrubina y sus metabolitos en el suero, la orina y las heces. La bilirrubina sérica puede ser cuantificada mediante (1) la "reacción diazo"; (2) cromatografía líquida de alta resolución (HPLC); (3) métodos oxidativos, enzimáticos y químicos; (4) espectrofotometría directa; y (5) métodos transcutáneos [15].

Desde su descubrimiento a finales del siglo XIX la reacción de la bilirrubina con ácido sulfanílico diazotado, conocida como la reacción diazo, ha sido la base de los métodos más ampliamente utilizados para medir la bilirrubina en suero [16]. En 1916 van den Bergh y Muller observaron que, en el suero de lactantes con ictericia, esta reacción era lenta y requería un acelerador para proceder, mientras que era rápida en bilis y en sueros adultos, sin adición de etanol. Esta particularidad condujo a los términos bilirrubina indirecta y directa, respectivamente [17]. La naturaleza química de las bilirrubinas directas e indirectas fue dilucidada en los años 50, cuando el uso de la cromatografía reveló tres fracciones de bilirrubina: bilirrubina no conjugada (fracción de reacción indirecta), monoglucurónido y diglucurónido de bilirrubina (fracciones de reacción directa) [18]. Existe una cuarta fracción que resulta de la unión covalente de la bilirrubina a proteína (δ-bilirrubina); esta es distinta del complejo bilirrubina-albúmina presente en el suero [1].

### Método diazo

La reacción de la bilirrubina con el reactivo diazo rinde dos azodipirroles coloreados (azopigmentos) (Figura 1D) que slbse pueden medir espectrofotométricamente, alrededor de 530 nm a pH neutros y ácidos, y a 598 nm a pH alcalino (ej. tras adición de tartrato alcalino). La reacción se acelera por el alcohol y una variedad de otros compuestos (ej. benzoato de sodio) que causan la disociación de BNC de la albúmina [19]. En presencia del ’acelerador’ se miden conjuntamente (bilirrubina total) tanto la BC (incluyendo la delta) como la no conjugada. En ausencia del acelerador, solo reacciona la BC (’directa’). La diferencia entre estas se considera una medida de la BNC (’indirecta’). Para la exactitud del método lo más importante es que no haya BNC que reaccione en el procedimiento directo.

El método diazo descrito por Jendrassik y Grof en 1938 [20], y luego modificado por Doumas y cols [21] da resultados para la bilirrubina total sérica que son reproducibles y veraces; en este el acelerador es una solución de cafeína y benzoato de sodio. Este método tiene una transferibilidad aceptable entre laboratorios y es actualmente el *método de referencia* [15, 21–23]. Su veracidad para medir la bilirrubina total y directa se ha evaluado mediante el uso de bilirrubina no conjugada y diglucurónido de bilirrubina auténticas, cuantificadas mediante resonancia magnética nuclear.

### Cromatografía

Se ha utilizado la cromatografía líquida de alto rendimiento para medir la bilirrubina total (BT) en suero tras la adición de las 4 fracciones o especies individuales mencionadas (no conjugada, mono y di-glucurónido, y delta-bilirrubina). Los valores de BT por HPLC se correlacionan adecuadamente con los del método Jendrassik-Grof [24]. El HPLC permitió esclarecer el tipo de bilirrubina que perdura tras la resolución de la patología hepática inicial (delta-bilirrubina, de vida media superior al resto de las fracciones). También ha ayudado a identificar y dilucidar la naturaleza de las formas de bilirrubina presentes en la sangre o que se forman tras la fototerapia, aunque esto añade poco o ningún valor clínico a las determinaciones habituales [25].

A pesar de sus cualidades, la cromatografía por HPLC no reúne las condiciones para ser el método de referencia [15]. Sus niveles de precisión y veracidad en la medición de los niveles de BT no son satisfactorios por una serie de razones: la calibración se realiza con BNC con la suposición no probada de que las otras tres fracciones de bilirrubina tienen absortividades molares idénticas a las del calibrador; los errores en la medición de las cuatro especies pueden ser acumulativos y dar lugar a un considerable error total; asimismo parte de la δ-bilirrubina puede perderse durante el pretratamiento de las muestras. Para el análisis clínico de rutina el método es demasiado laborioso, además de insensible a concentraciones de bilirrubina total inferiores a 1 mg/dL (17 μmol/L).

### Métodos oxidativos

La bilirrubina puede oxidarse por un compuesto químico (vanadato) o por la enzima bilirrubina oxidasa (EC1.3.3.5) a biliverdina, que se oxida posteriormente a productos morados y finalmente incoloros. La disminución concomitante en la absorbancia a 450–460 nm es proporcional a la concentración de bilirrubina. Con la bilirrubina oxidasa, la bilirrubina total se mide a pH cercano a 8, y la bilirrubina directa cerca de pH 4. A pH 10 la bilirrubina oxidasa oxida selectivamente la BC y un muy pequeño porcentaje de BNC, pero no la δ-bilirrubina [26]. El método debe calibrarse con BNC en suero humano.

### Espectrofotometría directa

Este método implica la medición de la absorbancia a 437 nm, la absorbancia máxima de bilirrubina. La interferencia causada por la hemoglobina se elimina mediante el análisis en un sistema de 2 componentes, midiendo la hemoglobina a otra longitud de onda y restando la porción correspondiente. La bilirrubina total también se puede determinar con el uso del cooxímetro que se encuentra en los instrumentos de gasometría: en estos el espectrofotómetro es capaz de medir la diferencia entre los espectros de bilirrubina y hemoglobina [23].

### Métodos transcutáneos

En los años 80 se comenzaron a fabricar instrumentos que aprovechaban la espectrofotometría directa para realizar estimaciones no invasivas de los niveles de bilirrubina circulantes. Su rendimiento ha llegado a niveles muy aceptables en comparación con el método diazo, con una dispersión de ± 2 mg/dL (34,21 mmol/L) [27]. Aunque las mediciones transcutáneas no sustituyen a las determinaciones de bilirrubina en el laboratorio, pueden tener utilidad como pruebas de detección: proporcionan información instantánea en la cabecera del paciente, evitan al neonato el trauma del pinchazo, y pueden reducir el número y el costo de las determinaciones de bilirrubina sérica. Además, ayudan a determinar cuándo es necesario extraer sangre y guiar tratamientos como fototerapia o exanguinotransfusión. Se han realizado varios trabajos y revisiones sobre la utilidad que las mediciones de la bilirrubina transcutánea tienen en el manejo de la hiperbilirrubinemia neonatal [27], [28].

### Bilirrubina en la orina

Solo la BC es soluble y es filtrada por el riñón, por lo que su presencia en la orina indica hiperbilirrubinemia conjugada. El método más comúnmente utilizado para detectar bilirrubina en la orina es la tira reactiva (elemental), la cual en la fracción dedicada está impregnada con un reactivo diazo (frecuentemente dicloroanilina diazotada o diclorobenzenodiazonio fluoruroborato). Los métodos de tira reactiva son capaces de detectar concentraciones de bilirrubina tan bajas como 0,5 mg/dL (9 μmol/L). Se requiere una muestra de orina fresca ya que la bilirrubina es inestable cuando se expone a la luz y a la temperatura ambiente, y puede oxidarse a biliverdina (diazo negativa) al pH normalmente ácido de la orina. Si la prueba se retrasa, la muestra debe protegerse de la luz y almacenarse a una temperatura de 2 a 8 °C durante no más de 24 horas. Este método no está exento de interferencias positivas (ácido ascórbico, nitritos) y negativas (sustancias que coloreen pardo/rojizo a la orina como fármacos o sus metabolitos; por ejemplo rifampicina) [29].

Es interesante señalar que la coexistencia en la misma tira reactiva de la medición del urobilinógeno permite conjeturar la naturaleza del trastorno en el metabolismo de la bilirrubina. La presencia de urobilinógeno aumentado, con bilirrubina aumentada o normal sugiere hemólisis aumentada o hepatopatías, con circulación enterohepática aumentada. En contraste, aumentos de bilirrubina con urobilinógeno normal apunta a una disminución de la secreción de BC al intestino, como en los casos de obstrucción biliar.

### Requerimientos analíticos y estatus de la medición de bilirrubina

Los requisitos para la medición varían levemente según su uso. Según las directivas CLIA, es necesario un 20% o menos de error total, y las especificaciones mínimas de consenso del Comité de Expertos Interdisciplinar de Especificaciones de la Calidad (CEIEC) de la SEQC/AEFA/AEBM y SEHH fijan en un 24% de variabilidad las especificaciones mínimas para la medición de bilirrubina en suero [30]. Otros objetivos, como los derivados de la variabilidad biológica sitúan el porcentaje de error aceptable (%CV) en un 11,3% [31]. Aunque en su inmensa mayoría los equipos y metodologías disponibles en el mercado cumplen con estos requisitos, es recomendable verificar su cumplimiento [5]. Subsisten algunas imprecisiones, sobre todo a concentraciones elevadas de bilirrubina [32] y en dependencia de la matriz (suero humano o bovino) de algunos de los materiales de control y calibración [33].

Los calibradores comerciales para los métodos de campo consisten en BNC y BC; esta última se incluye para calibrar métodos para bilirrubina directa. La matriz proteica de estos calibradores es suero humano, bovino, o una mezcla de ambos. La BNC en el suero humano reacciona completamente con el método de referencia y otros métodos diazo disponibles en los analizadores clínicos; sin embargo, su reacción en suero bovino de fuentes comerciales es incompleta e impredecible. Eso hace que la asignación de valores exactos de bilirrubina al material de calibración cuya base proteica es el suero bovino comercial sea virtualmente imposible [34]. En estudios realizados, en el suero humano la ditaurobilirrubina fue subestimada por dos de siete analizadores clínicos probados; ambos usaban calibradores basados en suero bovino. La ditaurobilirrubina en suero bovino comercial fue subestimada por todos los analizadores y por el método de referencia [33]. En consecuencia, la práctica de usar calibradores de bilirrubina con matriz de suero bovino debe abandonarse ya que compromete la exactitud de las mediciones de bilirrubina.

## Significado clínico

Las enfermedades o alteraciones que interfieren con el metabolismo de la bilirrubina pueden causar un aumento en su concentración sérica. El aumento de bilirrubina en la circulación (>1 mg/dL) [35] provoca su fijación en los tejidos, sobre todo en aquellos con mayor número de fibras elásticas (paladar, conjuntiva, etc.). Cuando se acumula de forma sustancial (generalmente por encima de 2,5 mg/dL) se observa una coloración amarillenta de las mucosas y de la piel, conocida como ictericia. La hiperbilirrubinemia por sí misma no es un trastorno de mal pronóstico [5], ya que existen mecanismos adecuados para su detoxificación (salvo en el neonato). Sin embargo, es un signo de perturbación en la producción o metabolismo de la bilirrubina.

Existen diferentes maneras de acercarse a la clasificación de las patologías que cursan con hiperbilirrubinemia. Según la localización del trastorno responsable de la hiperbilirrubinemia se pueden calificar en: pre-hepáticas, hepáticas, o post-hepáticas.

### Hiperbilirrubinemias pre-hepáticas

Serían aquellas hiperbilirrubinemias secundarias a un exceso de producción de bilirrubina. La causa más común es la hemólisis acelerada. Cuando el aumento del ritmo al que se produce la bilirrubina supera la capacidad de captación y excreción hepáticos, provoca el aumento del nivel sérico de BNC; la concentración de BC puede ser normal o estar ligeramente elevada. Habitualmente, no es difícil identificar la hemólisis como la causa de la hiperbilirrubinemia porque el paciente tendrá muchas otras manifestaciones de la enfermedad (anemia, aumento de reticulocitos, etc.) [1]. Dado que el aumento de bilirrubina no es debido a un daño hepático, no encontraremos alteraciones de las aminotransferasas, de la albúmina, ni de la actividad de protrombina.

### Hiperbilirrubinemias hepáticas

Serían aquellas patologías relacionadas directamente con el funcionamiento hepático. Pueden estar afectados los procesos de captación, metabolismo, conjugación y/o excreción de bilirrubina, por lo que se puede observar tanto un aumento de la BC, como de la BNC, o ambas.

Estas enfermedades se acompañan de una lesión hepática de intensidad variable que puede llegar a comprometer la función hepática, y pueden desarrollarse de un modo agudo o crónico. Especialmente en el daño hepatocelular agudo, la necrosis hepatocelular origina un aumento variable de las transaminasas. Las enfermedades hepatocelulares que pueden producir hiperbilirrubinemia son: hepatitis virales, alcohólica, esteatohepatitis metabólica, hepatitis tóxicas, enfermedad de Wilson, hemocromatosis, hepatitis autoinmune, déficit de alfa-1 antitripsina, hepatitis isquémica, síndrome de Budd-Chiari.

#### Con aumento de la bilirrubina no conjugada

Por desajustes en la captación hepática y/o en la conjugación, la BNC se acumula y aumenta en sangre; consecuentemente la BC disminuye. Esto también conlleva una disminución de la concentración de urobilinógeno que se puede apreciar en orina y en heces (Acolia). No existe aumento de bilirrubina en la orina (Coluria) dado que la BNC no es hidrosoluble y no es filtrada por el riñón.

Fármacos como la rifampicina, el cloranfenicol y el probenecid pueden producir hiperbilirrubinemia no conjugada al competir con el transportador que introduce la bilirrubina en el interior del hepatocito.

El aumento de BNC también está relacionado con defectos hereditarios que afectan la conjugación. De entre estos, el *Síndrome de Gilbert* es el más común en adultos y afecta del 3% al 10% de la población. La actividad de la UDPGT es baja. No suele requerir seguimiento ni tratamiento ya que es un trastorno benigno. No obstante, puede llevar a confusión durante el proceso de cribado de enfermedad hepática, y a menudo se diagnostica erróneamente como hepatitis crónica [3].

Cuando el defecto genético afecta directamente a la producción de la enzima se produce el *Síndrome de Crigler-Najjar* (SCN). El SCN tipo1 se caracteriza por un déficit completo del enzima, no mejora con la terapia de inducción con fenobarbital, y suele ser incompatible con la vida por la toxicidad neurológica que origina la deposición de bilirrubina en los ganglios basales y núcleos del tronco encefálico (kernícterus neonatal) [36]. En el SCN tipo2 el déficit enzimático es parcial y responde al fenobarbital y fototerapia, lo que permite a los enfermos alcanzar la edad adulta. La enfermedad es muy rara con una incidencia anual de 1/1.000.000 nacimientos [16].

Mención aparte merece la ictericia neonatal que se presenta en el 60% de los recién nacidos y en el 85% de los infantes pretérmino. Generalmente es fisiológica y está limitada a la primera semana posparto, es debida a la inmadurez hepática en la metabolización y excreción de bilirrubina. Si el aumento de BNC supera los 5 mg/dL/día existe un riesgo de desarrollo de kernícterus, especialmente en recién nacidos de bajo peso al nacer. Este síndrome se puede prevenir mediante fototerapia y, en caso extremo, transfusión sanguínea. Otras causas de hiperbilirrubinemia no conjugada en el neonato pueden ser la enfermedad hemolítica, o la hiperbilirrubinemia durante la lactancia materna y el hipotiroidismo, entre otras. La descripción de estas patologías no es objeto de este documento (ver referencias 27 y 36).

#### Con aumento de la bilirrubina conjugada

En estos casos los procesos de captación y conjugación funcionan adecuadamente, pero falla la excreción canalicular. Por este motivo aumenta la BC en el suero (por acumulación en el hígado), pero también la no conjugada [37]. Al igual que en la hiperbilirrubinemia no conjugada disminuye el urobilinógeno tanto en orina como en heces (Acolia), pero en estos casos se detecta coluria (aumento de bilirrubina en la orina) ya que la BC es hidrosoluble y es por tanto filtrada por el riñón.

Su aumento se puede deber a enfermedades hereditarias que afectan la excreción (*Síndromes de Dubin-Johnson y Rotor*) o a trastornos colestáticos intrahepáticos, hepatitis virales, alcohólicas, u otras hepatopatías [11] que incluyen diferentes tipos de entidades:

a) Trastornos propios de los conductillos biliares: Colangitis biliar primaria, colangitis esclerosante primaria de pequeño ducto, enfermedad de Caroli, enfermedad de injerto contra huésped, ductopenia del adulto, fármacos.
b) Trastornos infiltrativos: De origen infeccioso (tuberculosis, brucelosis, fiebre Q, sífilis, lepra), de origen sistémico (sarcoidosis, granulomatosis de Wegener, linfoma, amiloidosis), o formas tóxicas (alopurinol, sulfamidas).

La hiperbilirrubinemia conjugada tiene por tanto un importante grado de especificidad para daño hepático.

El *Síndrome de Dubin-Johnson* (SDJ) es un trastorno autosómico recesivo causado por mutaciones en el gen que codifica para ABCC2/MRP2, la proteína involucrada en la secreción de la BC hacia la bilis. Se caracteriza, desde el punto de vista clínico por una hiperbilirrubinemia crónica predominantemente conjugada, sin prurito; y desde el punto de vista histopatológico por depósitos de pigmentos de color marrón-negruzco (semejante a la melanina) en las células parenquimales hepáticas [38]. Las enzimas hepáticas no suelen estar alteradas, y aunque el nivel de excreción de coproporfirinas no aumenta, la relación normal de coproporfirina I a III se invierte. El pronóstico es benigno. Por su parte el *Síndrome de Rotor* es una rara enfermedad de hiperbilirrubinemia benigna similar al SDJ aunque sin pigmentos en el hígado. Las coproporfirinas totales en orina están elevadas, de las cuales aproximadamente dos tercios son de coproporfirina I.

Otros síndromes que cursan con aumento de la BC [>1,5 mg/dL (26 μmol/L)] son la hepatitis neonatal idiopática y la atresia biliar en el neonato. Estas entidades suelen ser difíciles de diagnosticar. La historia familiar puede ser útil en el diagnóstico de la deficiencia de α1-antitripsina, fibrosis quística, galactosemia, intolerancia hereditaria a la fructosa y tirosinosis.

Otros trastornos de base genética en los que existen alteraciones en los trasportadores biliares en la membrana canalicular del hepatocito son la colestasis del embarazo, colestasis intrahepática recurrente benigna y la colestasis intrahepática familiar progresiva.

Fármacos como los anticonceptivos orales y la ciclosporina pueden alterar la excreción de BC hacia el canalículo biliar.

### Hiperbilirrubinemias post-hepáticas

Se denomina colestasis a la detención del flujo biliar, que impide de forma parcial o total la llegada de bilis al duodeno. Esta detención del flujo biliar se acompaña del paso de los componentes de la bilis a la sangre. Es común que la colestasis se asocie con ictericia, pero hay situaciones en las que no existe retención de bilirrubina, por lo que colestasis e hiperbilirrubinemia no son términos equivalentes. En función de dónde se encuentre la detención del flujo biliar se habla de colestasis intra o extrahepática.

Esta hiperbilirrubinemia se suele acompañar de un aumento de las enzimas de colestasis (GGT y FA). No hay coloración en materia fecal (acolia), y hay coloración excesiva en orina (coluria) donde el urobilinógeno sin embargo estará disminuido [1]. La colestasis extrahepática puede estar causada por cualquier obstrucción física total o parcial de los ductos biliares a nivel extrahepático. Las causas más comunes incluyen: coledocolitiasis, compresiones extrínsecas de la vía biliar (neoplasia de páncreas, síndrome de Mirizzi), trastornos de los ductos biliares extrahepáticos propiamente dichos (colangiocarcinoma, colangitis esclerosante primaria o secundaria), e infecciones (CMV, parásitos).

## La bilirrubina como marcador diagnóstico y pronóstico

### En la patología hepática

Como ya hemos visto, la elevación de la bilirrubina puede obedecer a numerosas causas, por lo que es un marcador inespecífico de disfunción hepática. Tampoco un indicador *sensible* de daño hepático: en condiciones de normalidad el hígado es capaz de conjugar hasta dos veces la producción diaria de BNC, sin aumento de la concentración de bilirrubina total; asimismo, la capacidad de excreción de bilirrubina es 10 veces mayor que su producción [39]. No obstante, la hiperbilirrubinemia sigue siendo un marcador clásico de alteraciones hepáticas y biliares, y en ciertas patologías hepáticas tiene un valor pronóstico [3], [40].

En la fase hiperaguda del fallo hepático agudo, la concentración de bilirrubina es relativamente baja respecto al gran aumento de la concentración de aminotransferasas en plasma; sin embargo, en el periodo subagudo, la situación se invierte, con un gran aumento de la concentración de bilirrubina, que refleja la gradualidad del daño hepático [41]. El incremento de la concentración de bilirrubina en plasma es, en estos casos, un indicador de peor pronóstico y de mortalidad [42].

La hiperbilirrubinemia no tiene valor pronóstico en pacientes con hepatitis aguda producida por paracetamol, pero sí en la hepatitis de presentación aguda y subaguda producida por otras causas [43]. Una concentración de bilirrubina superior a 17,6 mg/dL es un criterio de derivación hospitalaria en pacientes con hepatitis aguda no producida por la ingesta de paracetamol [44].

La cirrosis hepática puede acompañarse de elevaciones progresivas de la bilirrubina. El aumento de la concentración de bilirrubina es un fenómeno relativamente tardío en las enfermedades hepáticas crónicas, reflejando una afectación importante de la función hepática [4].

En el deterioro agudo de una hepatopatía crónica (acute on chronic liver failure), el incremento de la concentración de bilirrubina favorece que ésta difunda libremente a la barrera hematoencefálica. Esta situación puede verse reforzada con la disminución de la concentración de albúmina, y la menor capacidad de transporte de bilirrubina [45]. La consecuencia es un efecto neurotóxico, con un incremento del grado de encefalopatía debida al aumento de la concentración de ion amonio. Una elevada concentración de bilirrubina es una variable independiente, asociada al riesgo de mortalidad en una semana [46]. Por otro lado, una concentración de bilirrubina ≥3,45 mg/dL, en la admisión hospitalaria de un hepatópata crónico, es predictivo de riesgo de mortalidad a corto plazo [38].

Las hepatopatías colestásicas se caracterizan por la interrupción del flujo biliar. Estas enfermedades, en sus fases avanzadas, cursan con aumento de la bilirrubinemia, generalmente conjugada [16], [43].

Es útil tener en cuenta que el aumento de bilirrubina en suero no se correlaciona necesariamente con el grado de función hepática, y que el marcador precoz y preciso de insuficiencia hepática que no debe faltar nunca en la evaluación de una enfermedad hepática aguda o crónica es el tiempo de protombina en su forma normalizada (INR).

### En la enfermedad cardiovascular

Desde mediados de los años noventa una serie de trabajos hallaron una sólida asociación inversa entre la concentración plasmática de bilirrubina y el riesgo de enfermedad arterial coronaria [47], [48], [49]. Esta asociación se hizo también evidente en la cohorte del estudio Framingham [50] y en una cohorte de pacientes con Síndrome de Gilbert [51] donde un discreto aumento de la concentración de bilirrubina se asoció a un menor riesgo de arterosclerosis. Esto indica que la bilirrubina es un factor protector para las enfermedades cardiovasculares, independiente de los factores de riesgo tradicionales.

Investigaciones clínicas recientes muestran que las concentraciones levemente elevadas de bilirrubina están asociadas con protección contra diversas enfermedades mediadas por estrés oxidativo, entre las cuales las patologías ateroscleróticas son las más relevantes clínicamente. Este tema ha sido objeto de una excelente revisión [52] que profundiza en el tema.

## Conclusiones

La bilirrubina forma parte del estudio básico de función hepática. Existen numerosas plataformas y métodos de medida, siendo el método diazo el de referencia. La muestra más utilizada es el suero o plasma, también es común su medición en orina; en esta última es particularmente importante el cumplimiento de las condiciones preanalíticas adecuadas.

A pesar de sus limitaciones en cuanto a sensibilidad y especificidad diagnósticas, la bilirrubina es un marcador de uso recurrente para la valoración de diversas patologías relacionadas con la función hepática y biliar. La medida de la bilirrubina total y conjugada permite aproximar el origen de la alteración; lo mismo es aplicable a las mediciones de bilirrubina y urobilinógeno en suero y orina. En el ámbito hospitalario, la medida de bilirrubina tiene un papel muy útil en el pronóstico de hepatopatías agudas y en el seguimiento de hepatopatías crónicas. Es necesario realizar una correcta interpretación de los resultados obtenidos en conjunción con la anamnesis del paciente, la magnitud de la alteración, y otros parámetros del laboratorio clínico.
